# Block-HPCT: Blockchain Enabled Digital Health Passports and Contact Tracing of Infectious Diseases like COVID-19

**DOI:** 10.3390/s22114256

**Published:** 2022-06-02

**Authors:** Md Mamunur Rashid, Piljoo Choi, Suk-Hwan Lee, Ki-Ryong Kwon

**Affiliations:** 1 Department of Artificial Intelligence Convergence, Pukyong National University, Busan 48513, Korea; mamunrashid.ete88@gmail.com (M.M.R.); pjchoi@pknu.ac.kr (P.C.); 2 Department of Computer Engineering, Donga University, Busan 49315, Korea; skylee@tu.ac.kr

**Keywords:** digital health passports, contact tracing, vaccination certificates, COVID-19, infectious disease, blockchain, smart contracts, IPFS, hyperledger fabric

## Abstract

Due to its significant global impact, both domestic and international efforts are underway to cure the infection and stop the COVID-19 virus from spreading further. In resource-limited environments, overwhelmed healthcare institutions and surveillance systems are struggling to cope with this epidemic, necessitating a specific strategic response. In this study, we looked into the COVID-19 situation and to establish trust, accountability, and transparency, we employed blockchain’s immutable and tamper-proof properties. We offered a smart contract (SC)-based solution (Block-HPCT) that has been successfully tested to preserve a digital health passport (DHP) for vaccine recipients; also, for contact tracing (CT) we employed proof of location concept, which aids in a swift and credible response directly from the appropriate healthcare authorities. To connect on-chain and off-chain data, trusted and registered oracles were integrated and to provide a double layer of security along with symmetric key encryption; both Interplanetary File System (IPFS) and Hyperledger Fabric were merged as storage center. We also provided a full description of the suggested solution’s system design, implementation, experiment results, and evaluation (privacy and cost analysis). As per the findings, the suggested approach performed satisfactorily across all significant assessment criteria, implying that it can lead the way for practical implementations and also can be used for similar types of situations where contact tracing of infectious can be crucial.

## 1. Introduction

The COVID-19 (SARS-CoV-2) epidemic can be regarded as the most significant global health disaster of recent history due to its extraordinary contagiousness. Its symptoms vary in severity from person to person, with the most common symptoms being exhaustion, cough, high temperature, and shortness of breath. However, not everyone who has the condition develops symptoms [1]. The spread of this pandemic has prompted many countries throughout the world to implement rigorous rules and procedures in an attempt to stem the virus’s rapid spread. These policies have had an impact on economic activity and a wide range of industries, including foreign travel, restaurants, and retail centers. Countries are beginning to loosen international travel restrictions and let travelers from select areas to cross the border as COVID-19 immunization [2] efforts continue. Furthermore, tourists from those countries are required to produce proof of vaccination or a negative COVID-19 test before crossing the border. So, it is time the world comes out with some concrete solutions to tackle this overall scenario.

To minimize its impact, concerned authorities have developed and implemented response strategies based on data relating to illnesses collected by regional units. Clinical diagnosis by health facilities and other specialist facilities is used to generate this information. The presence of several intermediates in this process, however, causes reporting delays, limiting hospitals and testing centers’ ability to respond quickly when illnesses are reported [3].

Emerging health technologies such as blockchain and artificial intelligence (AI) can be used with point-of-care diagnostics to assist patients who have been exposed to COVID-19. Blockchain is a distributed ledger that records transactions in a digital format. It entails the digital distribution of ledgers and consensus methods, as well as the elimination of any intermediary risks. In many scenarios, such as early identification of epidemics, medicine supply chain, and donations tracking, blockchain technology has already been recognized as a possible tool to battle against the pandemic [4]. In the same manner, the development of a blockchain network to share data about the epidemic will improve openness, accessibility, and transparency, reducing the possibility of data conflicts between varied sources, such as medical facilities or governments. Moreover, by making vaccine information publicly available, such a blockchain platform could enhance people’ participation in the immunization process [5].

In this paper, we introduced Block-HPCT (Health Passport and Contact Tracing), a blockchain-enabled digital health passport based on vaccination certificates and contact tracing to control the spread of COVID-19. As COVID-19-related immunity certifications have been criticized by a variety of sources, this study focused on vaccination certificates associated with an individual. These certificates are a digitized version of the paper-based certificates, which list a person’s vaccinations and the date they were given. By establishing a blockchain platform to analyze the performance needs of leveraging these certificates, our work is linked with this objective [6]. Another issue most of the authorities are facing currently is contract tracing, which can help with an efficient and automated algorithm for monitoring infected cases of COVID-19 and distinguishing linked contact cases as well as sending out notifications about infected people in the vicinity. 

Both digital health passports and contact tracing might be critical components in our battle against the pandemic, and a blockchain-based solution could be the best way forward for it. We offered an innovative blockchain-based approach that utilizes configurable smart contracts to implement functions and produce events that inform participants regarding medical updates, vaccination information, and requirements. Our approach can create trust and reduce frauds by removing third-party platforms, centralization, and identity theft. In our proposal, we suggest a hierarchy of trusted authorities, such as Ministry of Foreign Affairs, Ministry of Health, and Vaccination Centers to help maintain announcements and decision-making on course. In our attempt to remove centralization, we used the advantage of combining IPFS and Hyperledger Fabric in the form of distributed storage center. In addition to providing storage, they also provide double-layered security, which we discuss in detail in later segments.

Our research’s primary contributions can be best summed up as follows:

- We presented a blockchain-based approach that allows for COVID-19 vaccine- and test-takers to be identified as immunized/vaccinated. Without depending on any centralized authority, the proposed method uses the distributed blockchain ledger’s immutable events and logs;
- We also offered a solution for enabling and providing COVID-19 contact tracing that is completely decentralized, traceable, immutable, secure, and trustworthy;
- To securely store the patient/user’s medical, personal, and travel information, we combined InterPlanetary File System (IPFS) and Hyperledger Fabric as a storage center;
- To show the feasibility and trustworthiness of our solution, we conducted a cost, transaction time, and privacy analysis to demonstrate its effectiveness against similar proposed solutions;
- Our suggested solution is generic and can be readily adapted to meet the demands and standards of different kinds of contact tracing applications concentrating on infectious diseases.

The following is the breakdown of the structure of this article. The background and preliminaries on blockchain and related topics are presented in Section 2. Section 3 summarizes all the related works and studies relevant to our research topic. In Section 4, the proposed method and its implementation details are elaborately described. The smart contract deployment and validation in the proposed method are discussed in Section 5. Discussion on derived experiment results and their analysis along with the open challenges are highlighted in Section 6. Finally, in Section 7, the conclusions are drawn while mentioning future prospects and directions.

## 2. Background and Preliminaries

Due to serious security concerns, current solutions cannot be widely deployed, necessitating the development of a privacy-preserving COVID-19 contact tracking and digital health passport architecture. A variety of attacks on data can happen when they are being transferred or at rest and these attacks involve stealing crucial information and breaching patient privacy. Existing solution architectures are built on the assumption that all in-process stakeholders are trusted, and that only outsiders pose a threat to the systems. As a result, we have come up with a privacy-preserving solution for the application user that will prevent threats from both insiders and outsiders. Before going into the details of our solution, in the following parts of this section we briefly describe the background and prerequisites of relevant topics.

### 2.1. Blockchain

**Figure 1:**
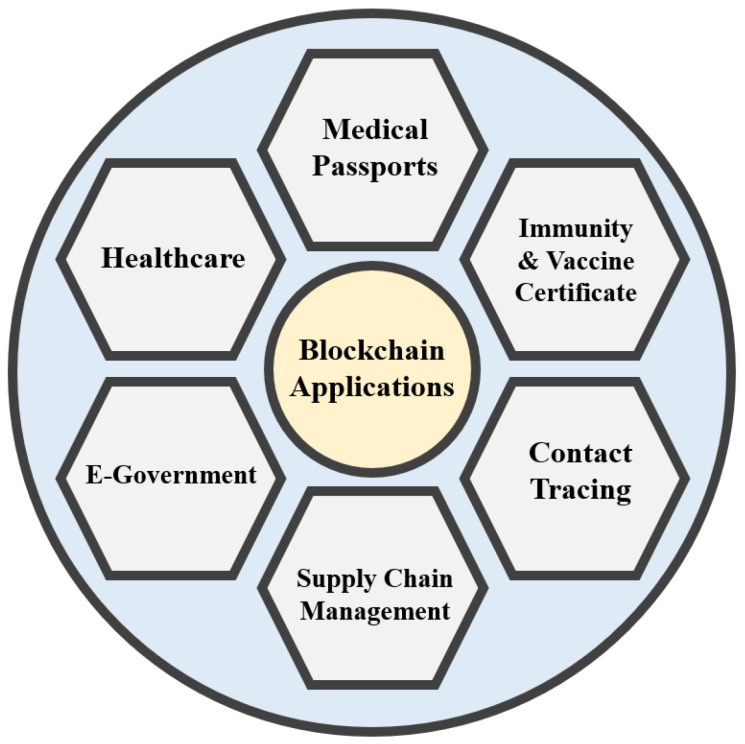
Blockchain-supported healthcare-related application areas in a pandemic situation.

Blockchain is a peer-to-peer distributed ledger that records transactions in a sequential, tamper-proof manner while keeping the application user/patient at the core of the healthcare ecosystem. It keeps track of an increasing number of data items that are grouped into data blocks. Following their inclusion into the blockchain, these blocks are cryptographically connected to the previous and future blocks. Individuals who are part of that system can share the transactions recorded on blockchains. Because each transaction is timestamped when it is entered into the ledger, it is immutable. Data sharing, access control, electronic records (EHRs, EMRs, and PHRs), supply chain, and other healthcare applications are all possible with blockchain technology [7]. Blockchain is used extensively in many areas to fight against the COVID-19 pandemic situation. In Figure 1, we can see some major areas of application and services that are currently getting explored [6,8,9,10,11,12].

### 2.2. Smart Contracts

Smart contracts (SC) are self-executing contracts in which the conditions of a multi-party agreement are written in code. They conduct all or part of the contract-related actions autonomously and generate associated proof that can be checked to prove the contract’s success [13]. Members must agree on how operations and their content are recorded on the blockchain, as well as the “if/when…then…” rules governing those transactions, in order to construct the terms. Users deposit the service charge into the contract in an environment that does not need a trusted third party, and the smart contract assists users in retrieving it. The service cost will be taken from the contract only when an acceptable outcome is obtained. In our proposal, we used several smart contracts to set up the conditions and rules as per our requirements, which we see in detail in Section 4.

### 2.3. IPFS and Hyperledger Fabric

The Interplanetary File System (IPFS) is an open source filesharing platform designed to distribute information in a peer-to-peer (P2P) manner across the web [14]. IPFS has the advantage over traditional cloud storage in that there is no central server and data are distributed and preserved around the globe. When a file is uploaded to the IPFS system, it is given a unique cryptographic hash string that may be used to retrieve the file. Due to block congestion and transaction costs, blockchain is not suited for storing huge data (video, music, etc.) in the practical applications. As a result, the encrypted file is stored in IPFS in our method.

Hyperledger Fabric is a distributed ledger application framework built on a scalable architecture that delivers high degrees of confidentiality, robustness, versatility, and scalability [15]. Because it allows for the transmission of private data, Hyperledger Fabric may be used as the underlying permissioned blockchain network [16]. It is extremely flexible and modular for a variety of use cases like tamper-proof audit trail, data access management, supply and origin tracking, commission management, and contract validity. In our case, we used Hyperledger Fabric with IPFS as data storage to protect them from any kind of security and privacy threats.

### 2.4. Digital Health Passports

A digital health passport is some sort of confirmation that a person has already been vaccinated or immunized against the COVID-19 virus in a digital format. The data is frequently displayed as a QR code on a smartphone or electronic wallet and can reveal if one individual is vaccinated or not. Especially with the advent of COVID-19, the industry has come together for the first time to support a digital alternative aimed to increase verifiability and avoid some of the constraints produced by the paper alternatives. The digital passport eliminates inefficiencies and alleviates dangers such as losing a traditional passport [17]. Both passengers and companies will benefit from the digital platform, which will help them establish a more efficient system for flying safely. In contrast to the reactive testing–tracing–isolating technique, it is a proactive effort aimed at preventing infected (and perhaps asymptomatic) people from entering the nation. This solution reduces the obstacles created by the reviewing individuals’ paper documents or answering a series of questions by providing a simple and effective approach to monitor vaccines and negative COVID-19 tests.

### 2.5. Vaccination Certificates

The introduction of COVID-19-related immunity certificates has raised interest among researchers, as noted in Section 3. However, as immunity certificates have been criticized by a variety of sources, this study focused on vaccination certificates associated with a vaccinated individual. Indeed, many governments throughout the world recently focused their concentration to develop a blockchain-based vaccination certification system. These certificates are a digitized version of the paper-based certificates, which list a person’s vaccination records and history. By establishing a blockchain platform to analyze the performance needs of leveraging these certificates, our work is linked with this objective [6].

### 2.6. Contact Tracing

The purpose involves identifying individuals who may have been in contact with an infected person and notifying them of the risk of infection is known as contact tracing [18]. In recent years, affected persons have been interviewed to determine who they have recently come into touch with using manual contact tracing. Manual contact tracing has the obvious disadvantages of being slow and needing appropriate labor. This issue can be addressed if the same objective can be accomplished by utilizing the vast array of communication and other digital technologies already available. The rapid growth of the world’s population and the expansion of overpopulation in countries necessitate the implementation of such a system. Furthermore, in most countries, the majority of the hospitals and medical administrations are missing a digital platform that can track the transmission of infection in real-time and provide accurate figures of the number of affected persons. 

The principal means through which the COVID-19 spreads is when a person comes into contact with someone who is sick (COVID-19 positive). A contact is defined by the World Health Organization (WHO) as:

- Spending more than 15 min within 1 m of a COVID-19 positive person;
- Direct/physical interaction with a COVID-19 confirmed patient;
- A person or a medical staff providing direct care to COVID-19 patients diagnosed without adhering to established SOPs (Standard Operating Procedures) or using PPE (personal protection equipment).

**Figure 2:**
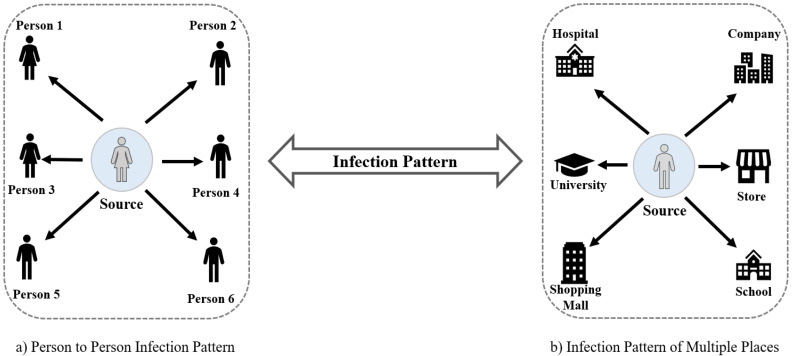
Representation of infection patterns: (**a**) infected persons and (**b**) infected places.

In Figure 2, the infection patterns considering two scenarios (infected peoples and places) are presented. These two scenarios can happen vice versa in any community and in situations like this, contact tracing becomes crucial.

## 3. Related Works

In this section, we discuss and summarize key works on the COVID-19 pandemic, with a focus on blockchain technologies to improve COVID-19 response.

Ting et al. [19] investigated how several cutting-edge technologies can aid in the prevention of the COVID-19 disease’s spread. The authors emphasized how the Internet of Things (IoT), big data, blockchain, and artificial intelligence (AI) might aid in the development of simulation models that forecast disease transmission. In terms of real-world applications, the authors cited the use of blockchain to trace pharmaceutical deliveries to patients’ doorsteps in China. Mashamba-Thompson [20] advocated using blockchain and artificial intelligence to enable COVID-19 self-testing. In this initiative, the emphasis is on showcasing the possible use of cutting-edge technologies to aid in the mitigation of COVID-19 dissemination. Technical implementations were not included in this study.

Torky et al. [21], on the other hand, provided a method for automatically detecting infected cases and estimating the COVID-19 infection risk in society using blockchain. To record the information and medical data of validated COVID-19 instances, the authors used blockchain’s decentralized property. Other technologies, such as an infection verification subsystem and a mass-surveillance system, rely on other technologies to discover infected instances.

Nguyen et al. [22] suggested a method for forecasting the propagation of the COVID-19 virus and other comparable outbreaks. They suggested combining AI and blockchain to process a vast amount of medical data with a complicated pattern. The report outlined a blockchain-based method for tracking donations and facilitating the healthcare supply chain. It did not, however, offer any technical details on the deployment.

Furthermore, Bansal et al. [23] showed how to create the immunity certificates using blockchain. To prevent the spread of misleading reports and information, the authors suggested using immutable blockchain technology. The proposed approach also sought to solve the issue of test-takers’ privacy and anonymity. However, the writers did not offer a design scheme or a strategy for achieving the proposal’s outcomes. Resiere et al. [24] presented a blockchain-based solution for reviving the Caribbean’s medical health system. As a result, they advocated using blockchain technology to establish medical collaboration and joint scientific research in order to combat the spread of COVID-19.

Frederiksen et al. [25] explored how to employ a distributed SSO protocol with proactive and adaptive security (PESTO) in digital health passports to improve the issuer’s security and the privacy of the users’ health data. They only offered a semi-formal representation of the whole protocol, claiming the need to abstract away issues such as digital identity validation and health authorities because they are all very implementation specific.

Kumar et al. [26] established a method for improving the deep recognition of a deep learning model for recognizing COVID-19 patients based on CT (Computed Tomography) slices. VGG16, VGG19, DenseNet, AlexNet, MobileNet, ResNet, and Capsule Network were compared to the authors’ proposed technique. Blockchain is being used in their studies to share data while protecting privacy.

The authors presented a contact tracing mechanism based on blockchain in ref. [27]. They emphasized the privacy difficulties raised by existing contact tracing apps as they use centralized servers and proposed a blockchain-based solution that might address the existing solutions’ privacy vulnerabilities. There were no development specifications or test results in their solution.

The Singapore government has also created the Singapore TraceTogether app [28], which is a Bluetooth-based contact tracing program. By transmitting short-distance Bluetooth signals between phones, this application instantly informs a person if they have been contaminated with COVID-19 through close contact with other TraceTogether users. After early testing on the Isle of Wight [29], a similar Bluetooth-based program called UK NHS Contact Tracing was scheduled to be deployed across England. Unfortunately, this App has yet to be finished since it contains several serious technical faults that prevent it from functioning properly.

With Bluetooth LE (Low Energy), Google/Apple Contact Tracing [30] takes a similar method. It differs from TraceTogether in terms of user privacy because the service provider does not have any access to the user’s true identity, making it privacy-preserving. Unfortunately, for contact matching and notifications, the user must use their centralized system, enhancing the risk of a potential threat on user privacy. The Chinese health code system [31] differs from the others in that it does not rely on Bluetooth or proximity sensing. It works by scanning the QR code connected with the user, which is based on relational cross-match. Because of the centralization of this mechanism, user privacy is not protected, and the information of the user is not hidden from the authorities.

BeepTrace [10], on the other hand, underlined the significance of incorporating blockchain into contact tracing to improve trust, openness, and privacy. As a result, their solution includes two types of blockchain networks: one for tracing and the other for notifications. When compared with alternative methods offered by the authors in terms of cost, security, and privacy, BeepTrace shows promise. However, it relies on the use of trusted third parties, such as the servers of the ’Geodata solvers’, as well as a Public Key Infrastructure (PKI).

The author presented a blockchain network called Bychain to avoid the usage of third intermediaries in ref. [32]. They changed the fields of the blockchain blocks they employ in the network to fit their block proposals and validation scheme. Short-range communication is also critical to their solution (SRC). The messages’ latency, power consumption, processing, and storage restrictions were all tested on the blockchain network design that had been established. Their primary goal is to put the newly developed Bychain to the test, as it only provides limited information on COVID-19 contact tracing data.

Garg et al. presented a unique application to aid social distancing [33]. The objective was to assist health officials in promoting social distancing by limiting the number of people in certain places. The blockchain is managed by various government entities. Citizens establish a wallet in which they obtain “movement passes”, or time-based tokens, which may be used but expire after a certain amount of time. This allows the authorities to limit the overall number of tokens distributed for a certain location during a specific time of day in order to limit the number of people in the neighborhood.

Furthermore, the study by Song et al. [34] provided a blockchain-based contact sharing of information and threat alerting system. Bluetooth was used as a short-range communication technology in their solution. Their technique focused on calculating the likelihood of a user contamination based on information supplied by the user or information shared by others.

Many European nations have already implemented their individual digital health passport solutions. Let us first consider the Danish corona passport [35], which is no longer necessary by Danish authorities, but is required for international travel. Corona passport is a user-friendly app that gathers information on vaccination, testing history, and immunity from prior infections in a single user-friendly solution and it only shows a few or no personal information about the individual. In Belgium, they have introduced another COVID-19 app named “GovApp” [36], which can work in foreign countries if phone has an internet connection. It merely saves the phone number of the user and nothing else. It can send alerts and notification about a COVID-19 test and whether the user came into contact with an infected person. It utilizes centralized storage, which can be accessed by central health authorities and is susceptible to a single point of failure.

“TousAntiCovid” [37] is another app introduced in France, which is capable of providing tracking COVID-19-positive patients and can generate travel certificates. The primary technological and operational decisions, as well as the centralized design, safeguard it from cyber-attack. The “CovPass-App” [38] developed in Germany is established on the “EU Digital COVID Certificate” and exhibits a QR code as confirmation of COVID-19 vaccination or recovery from infection. Like most of the solutions, this one is also based on a centralized storage system and provides less trust and transparency to the users. 

In conclusion, while several ideas and solutions for using cutting-edge technologies such as blockchain, Artificial Intelligence (AI), and IoT to facilitate COVID-19 digital health passport and contract tracing are discussed, most of the implemented solutions are either based on centralized servers or have very limited deployment results to support their claim. Furthermore, none of the aforementioned methods demonstrate a manner of preventing the propagation of the COVID-19 by utilizing blockchain technology directly. Blockchain is either recommended as a potential technology to restrict the dissemination of false information, or is joined by other technologies to provide a framework in the majority of articles. None of the studies offered or deployed a blockchain-based method for tracking and tracing COVID-19 test-takers via associated and authenticated vaccination certificates. In our research, we leveraged the blockchain technology to design a digital health passport and contract tracing solution which demonstrates its superiority over similar proposals discussed in this section.

## 4. Proposed Solution and Implementation

This section provides a full explanation of the suggested blockchain system’s design. Our system makes use of Ethereum smart contracts, as well as immutable records and trustworthy events. It aids in the tracking of patients for medical testing and travel records as well as vaccination status. In developing an identity for its entities, it relieves the burden on employers, government facilities, social and academic services, and transportation networks. It also aids in the containment and mitigation of the COVID-19 virus. As there are no centralized systems involved, the load that old centralized systems posed is alleviated. This adds a dimension of trust, transparency, and integrity to the equation. In later parts of this section, we describe the proposed Digital Health Passport and Contract Tracing solutions separately.

### 4.1. Digital Health Passport with Vaccination Certificates

**Figure 3:**
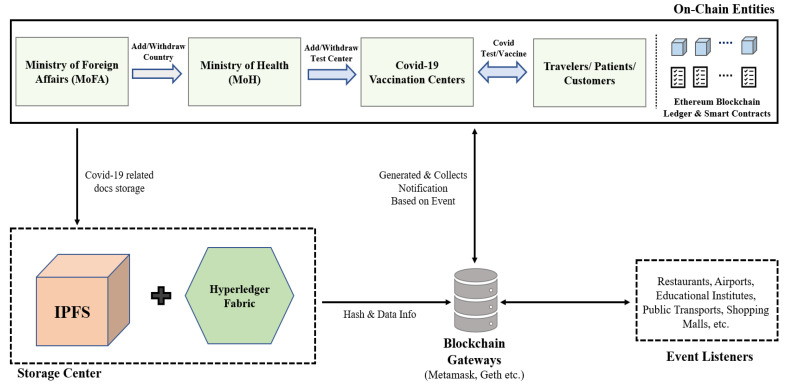
Proposed blockchain architecture for digital health passports with vaccination certificates.

As can be seen in Figure 3, we used blockchain technology to create a system that enables countries to communicate COVID-19 information, particularly vaccination and test-taking data, amongst them. It is evident from the figure that there are four major parts in our proposed architecture, namely: (i) On-Chain Entities, (ii) Distributed Storage Center, (iii) Blockchain Gateways, and (iv) Off-Chain entities. We describe the features of these parts in this section.

#### 4.1.1. On-Chain Entities

In our approach, we used four different types of smart contracts as on-chain participants: (i) the MoFA smart contract, (ii) the MoH smart contract, (iii) the Vaccination Center smart contract, and (iv) the Patient smart contract. The code is developed in the Solidity programming language [39], and the smart contracts are compiled and tested using the Remix IDE [40]. As we move forward to the later part of the paper, we describe the characteristics of these smart contracts, as well as other system sub-components.

- The MoFA Smart Contract

We designed our MoFA (Ministry of Foreign Affairs) smart contract for the purposes of adding the new eligible countries and removing ineligible countries from previously approved list. Only the owner of the MoFA contract has the authority to perform these actions. These two functions would therefore notify respective events to all of the parties involved, notifying them of the update and the time. There are many MoH smart contracts that can be linked to the MoFA smart contract.

B. The MoH Smart Contract

A COVID-19 vaccination or screening center that satisfies the requirements prescribed by a country’s Ministry of Health (MoH) is added to the MoH’s associated testing centers. If a COVID-19 vaccination center needs to be removed from the list of previously approved testing facilities, the MoH’s criteria could be applied. Only the owner of the MoH smart contract has authority to activate the function that adds or removes the authorized vaccination facility. The time of the addition or revocation of a vaccination center is published as an event in this function, and it is notified to all interested parties.

C. The Vaccination Center Smart Contract

Using this Smart Contract function, a COVID-19 vaccination center may publish notifications in the shape of events to alert the participating entities of every development. The notification might also include any new information regarding the patient. It could be about the projected time a COVID-19 vaccine results will be ready, or it could be about something relevant to vaccination. It can also announce whether any user is vaccinated or not through another function embedded in this smart contract. Only the patient’s Ethereum Address (EA) and the patient’s smart contract EA are logged in order to protect the patient’s privacy. The message also includes the moment when the outcome is aired.

D. The Patient Smart Contract

A patient can use this smart contract to update information stored on the storage center, as well as to perform the update function. Only the patient can use this function, which generates an event that indicates when an update occurred. Once the patient’s smart contract has been updated, it must be validated by a vaccination center associated with the MoH. The vaccination center EA, the patient’s EA, and the patient’s smart contract EA are all included in the event, as well as the time of the verification. This verification is vital to notify listeners that the update is authentic and has been approved by an authorized party.

#### 4.1.2. Distributed Storage Center for Data Confidentiality

We designed our storage center leveraging the combination of IPFS and Hyperledger Fabric to provide extra protection and security to the data. The cost of storing records relevant to COVID-19 screening, identification, vaccination, and travel on the main blockchain would be massive, where IPFS can surely help.

The data owner can upload files to IPFS, and then the file hashcode and encryption keys are moved to Hyperledger to be stored, according to our system architecture. Our system allows several parties to access the servers’ content while ensuring privacy [41]. A second form of security in Hyperledger Fabric, where the encrypted hash info (which will include the metadata of the stored files) and the encryption key will be maintained for increased safety and privacy, is also suggested in our solution. Metadata for a file can include information such as the recorded time and date, speed, related logs, Geolocation, device identifiers, and so on. This special attribute of metadata provides protection against any attempt to upload fake files or documents into the system as it differs from the original metadata. The key used to encrypt the uploaded data is only known by the data owner and only the permitted smart contract will have the hash information derived from the distributed storage center. Any interested party (academic institutions, travel agents, and transportation facilities) will require both the hashcode and the key to view/access the data stored in distributed storage. 

**Figure 4:**
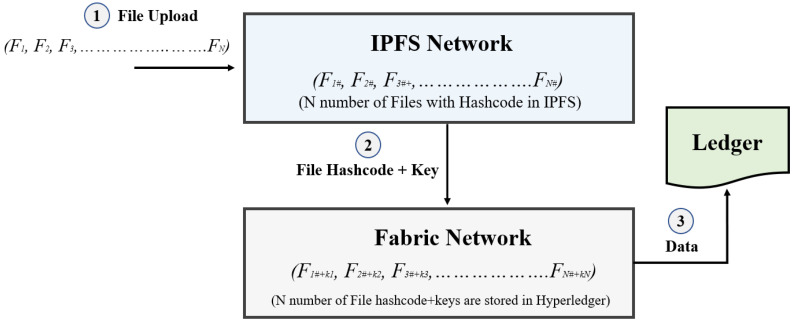
Storage of data with confidentiality using IPFS and Hyperledger Fabric.

As can be seen in Figure 4, in step 1 of our data storage process, COVID-19-related files are uploaded to IPFS network where using symmetric key the encryption happens. Then in Step 2, generated hashcodes and their respective keys are moved to the Fabric network and in step 3 the relevant data are stored in the ledger. As we already mentioned, any receiver who needs to access or retrieve the data stored in this storage will need both the hashcode and key. When a receiver requests data from the storage center, the data owner generates a new key with the receiver’s public key and own private key. The interested parties can access the data using the newly generated key and the hashcode after the verification from the smart contracts, as only the permitted smart contract possesses the hash information. In this way, our proposed approach will provide two layers of security, preserving data immutability and integrity. 

#### 4.1.3. Blockchain Gateways

One of the current major obstacles for the widespread adoption of blockchain technology and distributed ledger technology (DLT) in general is interoperability among these platforms where blockchain gateways can play a key role [42]. A blockchain gateway allows secure and private transfer of data assets and related information across blockchain networks and with event listeners and that is why they are accommodated in our proposed framework, as can be seen in Figure 3. Several blockchain clients are now in existence, including Metamask [43], Infura [44], Geth [45], and others. Their main intention is to provide data to the appropriate listeners in a secure way. This improves the solution’s security and decentralization. The gateways can send evidence of fraud to the blockchain network, and the malicious user can be penalized.

#### 4.1.4. Event Listeners

Event Listeners are entities interested in obtaining regulated events from the public blockchain and interact with the blockchain gateway to do so [46]. In our proposal, we considered restaurants, airports, shopping malls, educational institutes as the event listeners that can benefit from our suggested model. Smart contract events are public and accessible to everyone on the blockchain, reflecting the transparency, trust, and immutability features. Vaccine/test takers and associated vaccination centers are included as events in our solution. As a result, if a user needs access to a patient’s identity documents, they may utilize the *hash+key* to get the information they need. As COVID-19 is extremely infectious, it is critical for all relevant places where human interaction is unavoidable to guarantee COVID-19 protection. Users may need to access any off-chain papers that could be necessary for validation, such as passport information, travel history, COVID-19 test results or date, and other identifying documents stored in the storage center.

### 4.2. Contract Tracing

**Figure 5:**
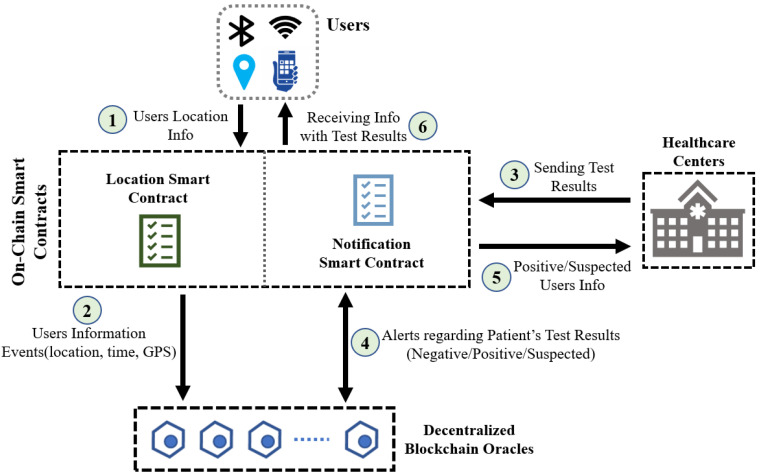
Proposed contract tracing solution using blockchain.

As shown in Figure 5, this section shows the system design of the COVID-19 contact tracing portion from our original proposal. In this solution, we also have four parts which are: (1) Users, who are responsible for sharing location info, (2) Ethereum Blockchain, where two smart contracts, namely, Location SC and Notification SC are designed, (3) Healthcare Centers, where patients usually go for testing purposes, and (4) Decentralized Blockchain Oracles, who maintain the connectivity of smart contracts with interested parties.

#### 4.2.1. Users

These users are responsible for sharing proof of locations [47] that includes relevant location info such as latitude, longitude, time and date. This information is fed towards the Location Smart Contract of the Ethereum blockchain. Users can use Bluetooth, mobile data, or Wi-Fi for communication. The distance between two users in close proximity is estimated using geo-location data shared between them. The proof of locations should be reported on-chain if the distance is less than 1.5 m. The distance between two geopoints can be obtained by utilizing their latitudes and longitudes to calculate the distance between two points on a sphere. To ensure the security of the user there, is a delay of 30 min before sending the location info to the blockchain ledger. 

To utilize the contact tracing application as well as blockchain capabilities, users need to be registered using their Ethereum Address (EA). Healthcare centers are the main authority to handle the whole registration process where every user EA is connected with their biometrics data. Additionally, enrolled users receive a digital health passport (discussed in detail earlier in this section) with all of their medical data. Users have the authority in disclosing their information to any kind of authority that seems appropriate.

#### 4.2.2. On-Chain Smart Contracts

Two on-chain smart contracts were incorporated in our suggested approach. Users’ locations are logged in ‘Location Smart Contract’ and all the alerts and notifications are controlled by ‘Notification Smart Contract’. Longitude, latitude, and time are all sent to be permanently recorded by Location SC. This smart contract can also send out events that are broadcasted to all oracles and participating entities, which ensures that the GPS coordinates will be available when contact tracing is required. 

On the other hand, after the COVID-19 test results are submitted by the healthcare facilities, the smart contract sends out several notifications/alerts which are controlled by the Notification SC. The issued alerts are then utilized to update the users’ on-chain profiles and notify the registered oracles of the developments. The COVID-19 alerts reported on-chain are used by the registered oracles to determine if a contact list is necessary depending on the alert types (negative/positive/suspected). The smart contract is encoded with the individuals’ EAs, and the results are sent out as suspicious notifications. These alerts are used by healthcare facilities to warn users that they may have been infected by a virus.

#### 4.2.3. Healthcare Centers 

Healthcare centers [48] that specialize in COVID-19 testing would send the results to the ‘Notification SC’, which might issue an alert depending on the results. They also expect a response from the Notification SC, which will assist them in determining who needs to be quarantined, tested, and treated if necessary. In response, the SC issues suspicion alerts based on the results of the registered oracles’ contact tracing algorithms.

#### 4.2.4. Decentralized Blockchain Oracles 

Blockchain oracles [49] connect blockchains to external systems, allowing smart contracts to run depending on real-world input and output. We deployed a group of registered oracles to connect with Ethereum smart contracts on-chain, ensuring that the blockchain has access to trustworthy and secure off-chain data. To handle the activities of the registered oracles we can use another smart contract. In response to the received alert from the ‘Notification SC’, the registered oracles run the contact tracing algorithm. Each registered oracle must respond within the timeframe set by the smart contract. Each registered oracle has a reputation that is impacted by how well it detects malicious or suspected users. To encourage other oracles to answer quickly, the winning oracle is rewarded with Ether.

## 5. Smart Contract Deployment and Validation

In consideration of the technologies incorporated and the design objectives stated in Section 4, the following section demonstrates the deployment results of our proposed solution. The technologies that were implemented were carefully chosen, resulting in the achievement of the intended security goals.

### 5.1. Details Results of Digital Health Passport Using Vaccination Certificates

In this subsection, we display the output from our coded smart contracts for an effective health passport.

#### 5.1.1. The MoFA Smart Contract 

**Figure 6:**
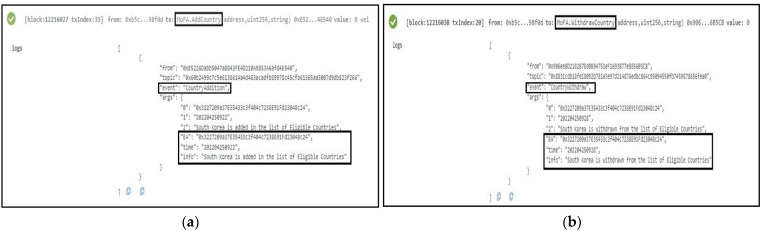
Deployment of MoFA Smart Contract. In (**a**), the *AddCountry* function is successfully deployed with Country’s EA, time, and info. In (**b**), Withdrawal of country is performed successfully by the *WithdrawCountry* function.

As we mentioned in our previous section, MoFA SC is responsible for handling the authenticated country addition and removal of unauthenticated countries. Two functions defined in SC, namely: *AddCountry* and *WithdrawCountry* are responsible to perform these jobs. All event listeners will be informed in case any new country is added or withdrawn to/from the list of approved countries. The specific time, date, and details info is accommodated in the SC as can be seen in Figure 6a,b.

#### 5.1.2. The MoH Smart Contract 

**Figure 7:**
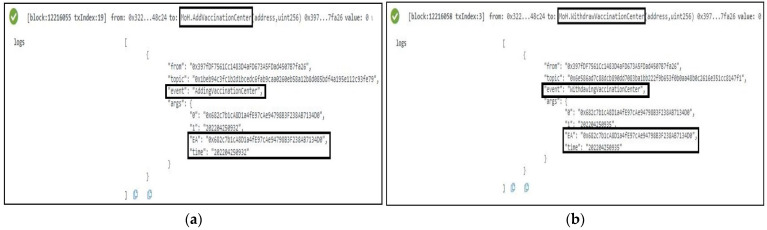
Successful deployment of MoH Smart Contract: (**a**) the addition of the vaccination center is completed; (**b**) withdrawal of a vaccination center is performed.

Vaccination center authentication is one of the most important aspects of our design as our suggested passport is dependent on that information. Addition or withdrawal of any vaccination center is handled exclusively by this SC through two functions, namely: *AddVaccinationCenter* and *WithdrawVaccinationCenter*. Only the owner of the MoH has the authority to perform these two functions and after execution relevant parties are informed accordingly regarding the events. As can be seen in Figure 7a,b, the MoH owner executes the events and the specific time and date are stamped on those events, which are totally temper-proof.

#### 5.1.3. The Vaccination Center Smart Contract 

**Figure 8:**
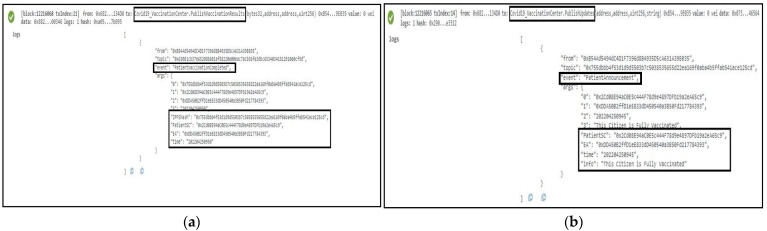
Logs of the deployment of Vaccination Center Smart Contract: (**a**) Vaccination results are published; (**b**) vaccination result is announced.

The function *PublishVaccinationResults* is called after the COVID-19 lab test vaccination results are ready to be collected. This function is successfully completed, resulting in the generation of an event that serves as a notification to all relevant stakeholders. The COVID-19 patient’s EA, SC address, time, and IPFS hash of the test results are also included in the event. Another function, *PublishUpdates*, was developed and tested to ensure that the event generated as an announcement notification contained all important details. The patient’s SC address, the time of the statement, the information that must be published, and the patient’s EA are among the details. Figure 8a,b show the logs and specifics of these two events.

#### 5.1.4. The Patient Smart Contract 

**Figure 9:**
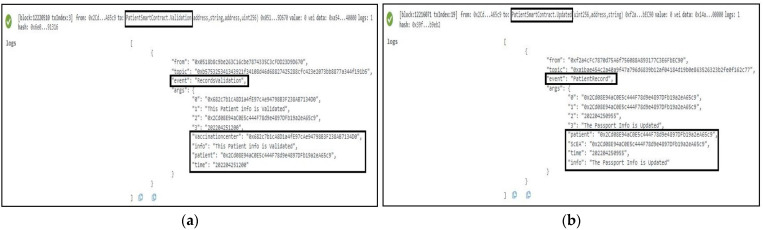
Patient Smart Contract deployed successfully: (**a**) the validation of the patient’s records is confirmed; (**b**) the passport information is updated with patient’s records.

The vaccination center must authorize any changes to the patient’s smart contract. As a result, as illustrated in Figure 9a, the owner of the vaccination center associated with the patients uses the *RecordsValidation* function to generate an event. The event notifies all participants of the validation time as well as the patient’s EA. It also provides notification information that indicates whether the patient’s information has been validated or not. Every time an update is reported using an event indicating the change, and the owner of this smart contract executes the *RecordUpdate* function. Figure 9b depicts the event, which includes the patient’s EA as well as the SC address, the creation time, and the updated information.

### 5.2. Details Experiment Results of Contact Tracing

The output details from our coded smart contracts for an effective contact tracing solution are displayed in this subsection.

**Figure 10:**
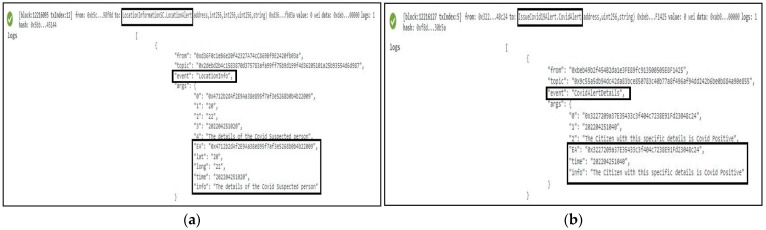
Logs representing deployment of the Contract Tracing smart contract. In (**a**), the location info of the user is depicted while in (**b**), the alert details of a COVID-19-positive patient’s info are accommodated.

Whenever a person is suspected of carrying the COVID-19 virus, we employ a function named *LocationAlert* to notify the citizens around him/her by triggering an event called “LocationInfo”. The latitude, longitude, user EA, time, and one display message containing the notification details were successfully sent as an event to all participating stakeholders as part of the immutable logs, as shown in Figure 10a. We also applied another function called *CovidAlert* that is exclusively responsible for the circulation of the alert details accommodating the patient’s EA, and time so that the specific user can take necessary precautions and measures to limit the spread of COVID-19. This function and logs are demonstrated in Figure 10b.

## 6. Discussion and Challenges

In this section, we outline the offered benefits of the suggested architecture while evaluating its viability in a real-world environment. We also discuss some of the blockchain technology’s constraints and open issues. 

### 6.1. Evaluation of the Solution in Terms of Blockchain Features

To evaluate our solution’s strengths, we use several key parameters, including security, privacy, authentication, access, scalability, interoperability, compliance, and cost analysis. We first explain a blockchain transaction authentication process to demonstrate its usability for our work and outline the offered benefits of the suggested architecture.

**Figure 11:**
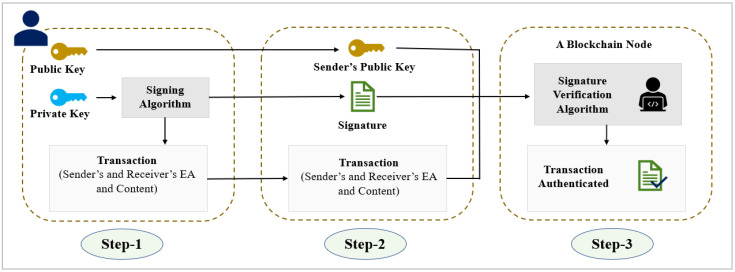
Transaction authentication process in a blockchain.

In Figure 11, we represent a transaction authentication process in a blockchain network for our proposed solution. In step 1, the sender generates a transaction and digitally transaction sender’s private key is signed combining with the signing algorithm. Then in step 2, the signed transaction is sent to the blockchain network along with sender’s public key. After that, in step 3, any blockchain node can authenticate the transaction by using the signature verification algorithm. In this way, blockchain authentication of any transaction maintains their integrity and authenticity.

Now, we describe how our solution enables the abovementioned blockchain parameters, which can be advantageous in a real-world setting from a contact tracing perspective in a pandemic situation.

#### 6.1.1. Security

Access is granted only to those who are authorized by the relevant authorities at each stage in our suggested architecture, and approval is provided after careful consideration. The agreement concerning which entity has access/permission to which resource preserves privacy. Only a pre-approved and trustworthy set of parties may access data stored on the storage center. Furthermore, because the content is homomorphically encrypted [50], the data remain private while in transit and at rest. Eavesdropping, spoofing, or any other sort of significant modification of source data is not feasible with the proposed architecture since the data are encrypted at the source. 

Furthermore, non-repudiation is a desired quality that assures that no entity can refute its actions. All on-chain transactions are registered using their credentials and the caller’s Ethereum address (EA), which are preserved in a tamper-proof method. As a result, no transaction on the chain is recorded without the caller’s information, such as the Ethereum and smart contract address. Because of these, the solution we proposed satisfies security and traceability goals.

#### 6.1.2. Scalability

Since our architecture is built on blockchain technology, bottlenecks and single points of failure in the proposed solution are avoided. The system’s decentralized nature also guarantees that no single person or group of people with power can alter or modify the ledger’s entries. This is particularly important in situations when the participants do not have complete trust in one another. Participants of the distributed ledger can dynamically expand or reduce the number of participating nodes in the ledger to control the workload produced by its users. Each node in the ledger is recognized as equivalent to other nodes, although certain nodes might play distinct roles in the blockchain network depending on how they participate. Certain nodes can preserve partial replicas of the chain, whereas others keep the entire chain, and still others just might validate transactions. Thus, members can pick what particular role they would like to play in the system by concentrating on the types of nodes they include in the chain, as well as scale their engagement in the solution by expanding or reducing the number of nodes in the public ledger that they include.

#### 6.1.3. Access Control and Authentication

The proposed framework incorporates technologies that are especially intended for authentication and access control compliance. It makes use of the capabilities of a permissioned blockchain [16] (Hyperledger Fabric in our proposal) to completely manage portions of the architecture’s access control regulations. Users may share their information with selected trustworthy entities for a brief duration and a specific set of resources without having to provide their usernames, passwords, or other critical security credentials, which is a specialty of this authentication and access control technique. This method may also be used for system security and inspection.

Because of the incorporated technologies, our solution allows people/patients to keep complete access and control over their data while also granting access to government authorities with whom they choose to share it. This allows government authorities, hospitals, physicians, and patients to securely interact and share information in real time. This is also great for medical research, as it makes it easier to conduct studies that will help us learn more about COVID-19.

#### 6.1.4. Interoperability

Our approach might be useful for effective surveillance and COVID-19 record administration, allowing government entities to overcome traditional data management issues and increase productivity significantly. Our proposed architecture avoids the necessity for time-consuming reporting procedures, which might lead to a single point of failure between the COVID-19 maintenance entities. As a result, the cooperation between healthcare entities increases, which enhances the quality and timeliness of COVID-19 administration. By design, IPFS and Hyperledger support solutions from several vendors, ensuring that the various solutions are interoperable. The suggested design may be turned into a sophisticated application that can be integrated with a COVID-19 bulletin system already in place, enabling instant integration and interoperability.

#### 6.1.5. Consent with GDPR

The suggested architecture makes use of smart contracts to acquire access to vaccination data. Smart contracts will allow the passport authority as well as other concerned agencies to retrieve the data. In terms of GDPR compliance, both the users and the healthcare administrators will be notified about this incident, ensuring that Article 5 [51] of the GDPR is followed.

Consent criteria can be developed and integrated into smart contracts as part of the proposed architecture. The permission will be acquired when the vaccine recipient is registered with the vaccination authority. This method will ensure that our proposed system complies with Article 7 [52] of the GDPR.

#### 6.1.6. Compatibility with DApps

As our solution is built using Ethereum smart contracts, it is compatible with the Decentralized Applications (DApps) [53]. In contact tracing, the DApp users can trigger the proof of locations using their phones which are stored on-chain and protected through smart contracts. DApps can be accessed using browsers on smartphones by users with different operating systems. DApps provide certain benefits over regular apps, such as censorship resistance. Unlike standard apps, DApps have no single point of failure, making them more resistant to threats and guarantee almost zero downtime. DApps are open access has no single entity ownership or control, which helps increase trust among its users. Users from all over the world can effortlessly use DApps, and when the users move from one country to another, they will not need access to different portals or platforms.

### 6.2. Experiment Result Analysis 

In this subsection, we evaluate our experiment result from various performance parameters, such as cost analysis and transaction time of the smart contracts.

#### 6.2.1. Cost and Transaction Analysis

The cost and transaction analysis of the Ethereum smart contract code and function calls is presented in this subsection. Remix IDE is a simple tool for estimating gas costs for deployment and transactions, which are the two most common forms of gas costs. Every transaction on the blockchain is subject to a fee. The fee in Ethereum is calculated using the gas price (Gwei), which is the price per unit to calculate the transaction cost. To be relevant and effective, the coded functions must be deployed as promptly as feasible at the lowest possible cost. 

Table 1 displays the gas prices and transaction times for the various smart contract functions. It is worth noting that gas prices are constantly fluctuating due to network congestion. Both the transaction cost and time depend on the number of attributes accommodated in a specific contract event/function. As can be seen from the table, all the functions of our designed smart contract are cost-effective and the transaction time of those functions is extremely small.

#### 6.2.2. Security Analysis of Smart Contracts

As mentioned previously, the smart contract is developed using Remix IDE, which includes some code debugging and run-time error alerts. However, they are insufficient to generate confidence in the integrity of smart contracts. They should be tested for reentrancy issues, loops, and other typical vulnerabilities that render them particularly vulnerable to malicious users. Therefore, the constructed Ethereum smart contracts for Block-HPCT are assessed using specialized tools to uncover any code vulnerabilities. At first, we used SmartCheck [54], which analyzes the code for reentrancy, Denial of Service (DoS), infinite loops, and malicious libraries, among other faults and vulnerabilities. 

**Figure 12:**
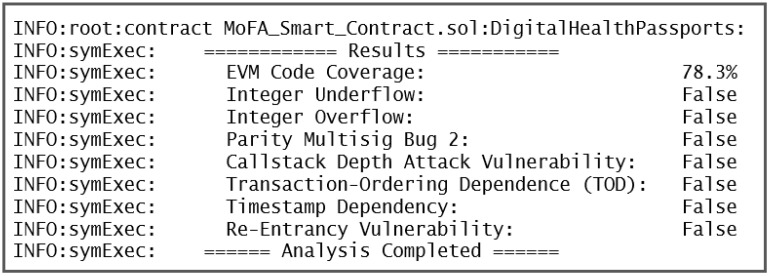
Vulnerability Analysis of Smart Contract using OYENTE.

After that, in the second phase, another static security analysis tool (OYENTE) for Solidity smart contracts was used to identify security vulnerabilities. Transaction ordering dependence, Callstack depth attack, Reentrancy vulnerability, Timestamp dependency, and unhandled exceptions are among issues that the OYENTE tool examines. OYENTE produces a summary report after evaluating the smart contract, as shown in Figure 12. It is evident from the Figure that our code is free of these vulnerabilities.

### 6.3. Comparison with Existing Solutions

One of the key differences of our solution was the integration of IPFS and Hyperledger Fabric so that together they can be used as one distributed storage center. Most of the solutions focused on a centralized server and when they chose a distributed storage, they opted for only IPFS. Although IPFS has its own advantage, using Hyperledger Fabric in combination with IPFS is more beneficial as we described in Section 4. This design effectively takes away a lot of burden from the on-chain storage system as all the documents and evidence material can be kept in distributed storage. They effectively provide a two-layered data security by generating hashcode and also using a symmetric key.

Another unique aspect of our solution was that we tried to leverage both digital health passport and contact tracing features in our solution. There are many solutions which we highlighted in the related work section that tried to address these issues separately, but in our solution, we accommodated both features that can be instrumental in curbing infectious disease from spreading at a massive scale.

Furthermore, one of the biggest drawbacks of blockchain technology is transaction speed. IPFS and Hyperledger Fabric can handle data at a considerably faster speed than traditional blockchain while providing better reliability. As IPFS uses content-based addressing and not location-based addressing, the data can be accessed using the cryptographic hash regardless when and where it was uploaded. We also can generally avoid acquiring a lot of redundant data because of this content-based addressing.

We understand it is a difficult job to compare the required resources for each system because of the architectural differences, and there are rarely any numerical performance parameters to measure performances of these kinds of solutions. We put together seven matrices for the comparison among contract tracing solutions, namely: technology, power usage, coverage, technology security, user cost, server and transaction cost, and user privacy and security. 

In Table 2, we compare Block-HPCT with other methods in terms of cost and privacy parameters. In terms of cost analysis, our approach delivered favorable results because the user cost of using location sharing technology/apps is very low, while the server and transaction costs of storing data and executing smart contracts are moderate. Table 1 analyzes user privacy and security by examining the blockchain security feature and privacy management beyond the user’s premises. The comparison table indicates that Block-HPCT has the advantage of reduced user end costs and higher security performance with stronger privacy protections.

### 6.4. Generalization in Real-World Settings

The suggested approach can help governments and healthcare organizations make crucial decisions based on transparent blockchain data, such as infection dynamics, infection identification, and contact tracing, which can help people safely participate in social events and institutions using the immutable logs. The preservation of user privacy will enhance the uptake of digital contact tracing amongst citizens, making it critical to win the battle against COVID-19. 

Our method is based on the notion of a medical passport, which precisely captures the symptoms of a disease type using medical documents provided by the patient and the healthcare organization. The distributed storage center (IPFS and Hyperledger Fabric) stores medical records and other forms of personal information.

Only the hash is saved in the patient’s contract, which functions as a medical passport and may thus be used to treat any ailment. Clinical testing facilities must also be associated with the appropriate authorities, such as the Ministry of Health, for any ailment that has to be traced, where both health passport and contact tracing can be useful.

Most immigration as well as travel locations today require a medical test and vaccination certificate before travel. Using smart contracts and immutable logs, information in travelers’ health passports can be easily traced and verified. Because of this solution’s compatibility with Decentralized Apps (DApps), it can be accommodated worldwide by all the nations with ease. Our solution can be used as a general framework for any type of infectious disease tracing as well as a profile for vaccine/test takers, including their travel and health information as well as vaccination records, due to its adaptability. 

### 6.5. Open Challenges

In this part, we go through some open challenges with blockchain technology before diving into some specific considerations for the COVID-19 epidemic. 

Even though blockchain is a revolutionary technology, certain difficulties have yet to be answered and require additional research. The most important is associated with blockchain platforms’ throughput, which may be insufficient for specific applications and is determined by the number of participating nodes in the protocol and the number of transactions created by them [9]. 

Transaction acceptance latency is another issue, which is determined by the time required to verify a block. New consensus methods have been created to address these issues, and are currently being researched.

Another challenge is that some sophisticated mass surveillance cyberattacks may be carried out if the attacker employs gadgets (behaving like ordinary smartphones). To overcome this challenge, combining advanced cryptographic methodologies with blockchain, such as Diffie Hellman [55] or Zero-Knowledge Proofs [56], may provide greater attack resilience and transparency.

The trade-off involving data auditability and privacy protection in another concern regarding blockchain. Although blockchain can help with GDPR compliance, there is still a concern that it violates Article 17 [57] since data cannot be removed from the blockchain.

Like our solution, several location-based systems take advantage of the Proof-of-location method, which verifies a user’s presence at a specific location at a certain time, with witnesses being WiFi access points or other devices. However, in order for this approach to be truly practicable, a rewarding or incentive mechanism need to be implemented, as it is unreasonable to expect these users/devices to willingly give up some of their resources, such as bandwidth, to implement Proof-of-Location procedures. 

The management of vaccination certificates, which can cause disparities in the population, requires special care. The certificates are likely to be handled by government agencies, which might lead to corrupt practices and prejudice against a section of citizens [58]. 

## 7. Conclusions

To prevent the spread of COVID-19, we proposed a decentralized blockchain-based digital health passport system (Block-HPCT) with vaccination certificates where contact tracing features are also accommodated. Our proposed solution utilizes Ethereum smart contracts to handle the guidelines and regulations of the on-chain entities. A unique concept of using IPFS and Hyperledger Fabric together as a distributed storage center was introduced, which ensures benefits in the form of security and confidentiality. To fulfil the purpose of contact tracing, decentralized blockchain oracles are used in conjunction with the Ethereum smart contract to handle the user’s location and generate notification/alert information. The integration of smart contracts protects users’ privacy and data ownership while also providing efficient access methods for different services. We also assessed the suggested method against similar solutions employing cost, transaction and security analysis, and found it to be inexpensive, fast, practical, and safe. The proposed method also assures privacy and can be simply integrated into other forms of contact tracing applications according to their criteria and requirements with minor modifications. 

With the goal of using the blockchain as a standard choice for emergency healthcare employment, to address the COVID-19 pandemic, the blockchain-based deployment should be upgraded to address issues mentioned in previous subsection. In the context of healthcare, for example, scalable and portable blockchain implementation are required to reduce data verification time and transaction delay. Accordingly, enriching blockchain through the use of new security measures should also be explored. Deep learning and other artificial intelligence techniques can help Block-HPCT become smarter and more efficient at identifying COVID-19 cases and forecasting epidemics. To achieve a solid healthcare infrastructure, blockchain can be integrated with other existing technologies as well to achieve satisfactory performance in resolving challenges linked with the COVID-19 pandemic. 

## Figures and Tables

**Table 1:** Smart contracts cost and transaction analysis.

Functions	Gas Price (Gwei)	Transaction Cost (Gas)	Transaction Fee (Ether)	Transaction Time (Seconds)
*AddCountry*	25.55	28.418	0.000612	<15
*WithdrawCountry*	25.76	28.468	0.000616	<15
*AddVaccinationCenter*	24.36	25.383	0.000728	<15
*WithdrawVaccinationCenter*	24.32	25.405	0.000776	<15
*PublishVaccinationResults*	22.26	26.947	0.000693	<15
*PublishUpdates*	23.76	28.251	0.000748	<15
*RecordsValidation*	25.54	27.652	0.000825	<15
*RecordUpdate*	25.18	29.134	0.000847	<15
*LocationAlert*	24.87	29.236	0.000795	<15
*CovidAlert*	24.59	28.420	0.000797	<15

**Table 2:** Comparison among different contract tracing solutions.

Solutions	Technology	Power Usage	Coverage	Technology Security	User Cost	Server Cost	User Privacy and Security
Singapore TraceTogether [28]	Bluetooth	High	Low	Weak	Medium	Low	Subpar
UK NHS [29]	Bluetooth	High	Low	Weak	Medium	Low	Weak
Google/Apple [30]	Bluetooth	High	Low	Weak	Medium	Low	Strong
Chinese Health Code [31]	GPS/QR-Code	Low	High	Medium	Low	Medium	Weak
ByChain [32]	Bluetooth, GPS	Medium	High	Strong	Low	High	Strong
BeepTrace [10]	Bluetooth, GPS, Cellular, WiFi	Medium	High	Strong	Low	High	Strong
Block-HPCT (Our Suggestion)	Bluetooth, GPS, Cellular, WiFi	Medium	High	Strong	Low	Medium	Strong

## Data Availability

Not applicable.
